# Mechanistic Characterization of the Pharmacological Profile of HS-731, a Peripherally Acting Opioid Analgesic, at the µ-, δ-, κ-Opioid and Nociceptin Receptors

**DOI:** 10.3390/molecules27030919

**Published:** 2022-01-28

**Authors:** Kristina Puls, Helmut Schmidhammer, Gerhard Wolber, Mariana Spetea

**Affiliations:** 1 Department of Pharmaceutical Chemistry, Institute of Pharmcy, Freie Universität Berlin, Königin-Luise-Str. 2+4, D-14195 Berlin, Germany; kristina.puls@fu-berlin.de; 2 Department of Pharmaceutical Chemistry, Institute of Pharmacy and Center for Molecular Biosciences Innsbruck (CMBI), University of Innsbruck, Innrain 80-82, 6020 Innsbruck, Austria; helmut.schmidhammer@uibk.ac.at

**Keywords:** GPCR, opioid receptor, HS-731, peripheral opioid agonist, analgesia, binding, selectivity, molecular docking, molecular dynamics simulations

## Abstract

Accumulated preclinical and clinical data show that peripheral restricted opioids provide pain relief with reduced side effects. The peripherally acting opioid analgesic HS-731 is a potent dual μ-/δ-opioid receptor (MOR/DOR) full agonist, and a weak, partial agonist at the κ-opioid receptor (KOR). However, its binding mode at the opioid receptors remains elusive. Here, we present a comprehensive in silico evaluation of HS-731 binding at all opioid receptors. We provide insights into dynamic interaction patterns explaining the different binding and activity of HS-731 on the opioid receptors. For this purpose, we conducted docking, performed molecular dynamics (MD) simulations and generated dynamic pharmacophores (dynophores). Our results highlight two residues important for HS-731 recognition at the classical opioid receptors (MOR, DOR and KOR), particular the conserved residue 5.39 (K) and the non-conserved residue 6.58 (MOR: K, DOR: W and KOR: E). Furthermore, we assume a salt bridge between the transmembrane helices (TM) 5 and 6 via K227^5.39^ and E297^6.58^ to be responsible for the partial agonism of HS-731 at the KOR. Additionally, we experimentally demonstrated the absence of affinity of HS-731 to the nociceptin/orphanin FQ peptide (NOP) receptor. We consider the morphinan phenol Y130^3.33^ responsible for this affinity lack. Y130^3.33^ points deep into the NOP receptor binding pocket preventing HS-731 binding to the orthosteric binding pocket. These findings provide significant structural insights into HS-731 interaction pattern with the opioid receptors that are important for understanding the pharmacology of this peripheral opioid analgesic.

## 1. Introduction

Opioid receptors are membrane-bound receptors belonging to the family of G protein-coupled receptors (GPCRs) [1]. There are four opioid receptor subtypes, including the three classical opioid receptors, µ (MOR), δ (DOR) and κ (KOR), and the more recently discovered nociceptin/orphanin FQ peptide (NOP) receptor [1]. The central role of the opioid system (opioid receptors and their endogenous and exogenous ligands) in pain treatment has been long recognized, with activation of each opioid receptor subtype leading to pain relief [2,3]. Because of their therapeutic relevance, the opioid receptors are among the few GPCRs determined in different activation states [4].

The most common strategy for the treatment of severe pain is by targeting the MOR [2,3,5]. Clinically used MOR agonists (e.g., morphine, oxycodone and fentanyl) are capable of producing potent and effective analgesia, but they also cause unwanted and numerous side effects, such as respiratory depression, constipation, sedation, nausea, tolerance, dependence and addiction [2,3,4]. Opioid misuse and opioid-induced overdoses and death have become a global medical and socioeconomical issue leading to the ongoing opioid epidemic [6,7]. Recently, it was reported that the overdose deaths from opioids was increased to 56,064 in 2020 in the USA [8]. Therefore, the development of safer analgesics with lower or no abuse liability and other undesirable side effects is highly needed [9,10,11]. Diverse approaches in the design of safer analgesics include targeting multiple receptors simultaneously (bi- and multifunctional ligands) [11,12,13], functional selectivity at GPCRs (biased agonists) [11,14,15,16] and peripheralization of opioid receptor agonists [2,11,17,18].

Opioid receptors are expressed in the central and peripheral nervous systems (CNS and PNS), and various non-neuronal tissues (immune, neuroendocrine and ectodermal cells) [2,3,19,20]. Preclinical and clinical studies have shown that selective activation of peripheral opioid receptors leads to effective pain relief and reduced CNS-mediated side effects [2,17,18,21,22,23]. Increasing the hydrophilicity of opioids to limit their access to the CNS, and thus to minimize the incidence of undesirable CNS effects comprises diverse chemical modifications, such as incorporation of quaternary or amphiphilic molecules, which contain hydrophilic and hydrophobic components, pH-sensitive activation of analgesic compounds and synthesis of peptide-derived analgesics. The goal of achieving analgesia while avoiding CNS penetration has focused on both small molecules and peptides [2,17,18,21,22,24].

Among the first generation of peripherally restricted opioid compounds was the MOR agonist loperamide [25] (clinically used in the control of diarrhea), which is completely excluded from the CNS by the action of P-glycoprotein [18]. Asimadoline [26], an amphiphilic molecule, was the first peripherally selective agonist with activity at the KOR evaluated in humans for the treatment of peripheral pain. Unfortunately, asimadoline did not achieve clinically relevant efficacy at doses that lacked CNS adverse effects [17,21]. Through computer simulations at low pH, the fluorinated fentanyl analogue, NFEEP, was identified as a potent antinociceptive activating specifically the MOR in acidified peripheral tissues and to lack the typical opioid side effects in animals [18,21,27]. Peripheral restriction can also be achieved with peptidic agonists that produce analgesia by activating the MOR or KOR in the periphery [17,22,28]. The most advanced peripherally restricted KOR agonist under clinical development for acute postoperative pain and chronic pain is the tetrapeptide CR845 (also known as difelikefalin) [17,22].

**Figure 1:**
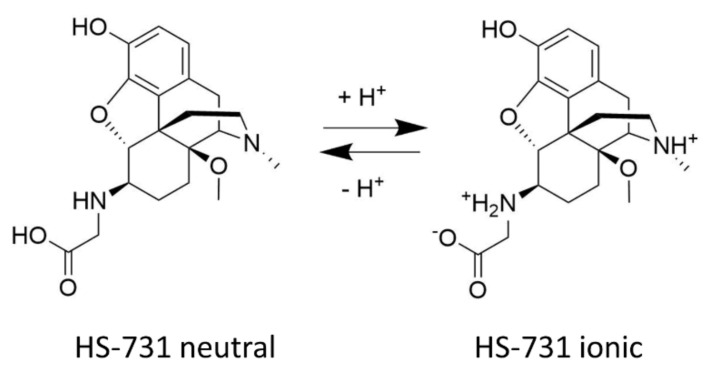
Structure of HS-731 and the acid-base equilibrium under physiological conditions.

Chemical and pharmacological work from our laboratory in the field of peripheral opioid analgesics from the class of opioid morphinans targeted the attachment of amino acid residues and dipeptides at the C6 position of the centrally acting MOR agonist 14-*O*-methyloxymorphone [29,30,31,32,33,34,35,36,37]. It was established that inclusion of an ionizable group, such as amino acid residues and sulfate conjugates, in morphinans leads to increased hydrophylicity and consequently reduced penetration into the CNS, by having greater selectivity towards peripheral tissues [31,32,34,35,37,38,39,40]. Inclusion of an ionizable group, such as amino acid residues, leads to increased hydrophylicity and consequently reduced penetration into the CNS, by having greater selectivity towards peripheral tissues. Several zwitterionic analogues were profiled as very potent MOR/DOR agonists producing antinociception after systemic administration in various pain models in rodents (mice and rats) via activation of peripheral opioid receptors [37]. A prominent representative of the series is HS-731, the 6β-glycine substituted derivative of 14-*O*-methyloxymorphone (Figure 1) [29], showing high affinity, potent and full agonism at the MOR and DOR, and a weak, partial agonism at the KOR (Table 1). In addition, HS-731 has been demonstrated to effectively induce peripheral opioid antinociception in a multitude of pain conditions, including acute nociception (tail-flick test) [31], visceral pain (acetic acid-induced writhing assay) [34,37], inflammatory pain (formalin test [31,33] and carrageenan-induced hyperalgesia [32]), neuropathic pain (sciatic nerve ligation) [33] and migraine pain (eye-wiping trigeminal nociceptive test) [36] in rodents. In acute thermal nociception, HS-731 was up to 200-fold more potent than morphine and had similar potencies to fentanyl when given systemically subcutaneous (s.c.), with considerably long-lasting antinociceptive effects. A significant and prolonged duration of the antinociceptive effect (up to 4 h) with a peripheral site of action was shown after oral administration of HS-731 to rats with carrageenan-induced inflammatory pain [32]. Recent data were reported on the absence of analgesic tolerance for HS-731 in rats upon chronic s.c. treatment for 14 days [23].

In the present study, we present the first mechanistic in silico investigation of the binding mode and interaction mechanisms of HS-731 to the three classical opioid receptors and rationalize why HS-731 does not bind to the NOP receptor.

## 2. Results and Discussion

### 2.1. HS-731 Shows No Specific Binding to the NOP Receptor

**Figure 2:**
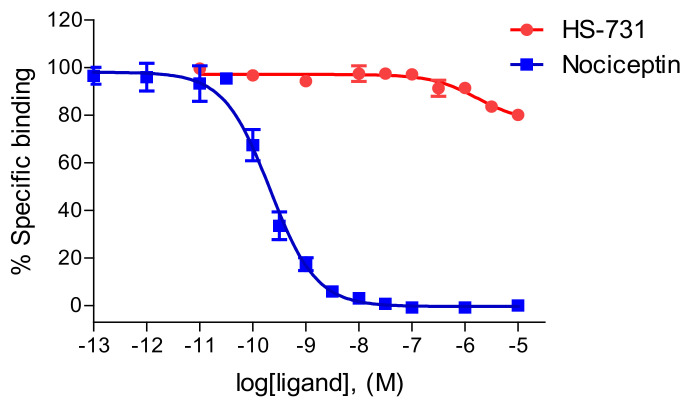
Binding curves of HS-731 to the human NOP receptor determined in the competitive radioligand binding assay. Concentration-dependent inhibition by HS-731 and nociceptin of [^3^H]nociceptin binding to membranes from CHO cells stably expressing the human NOP receptor. Values are means ± SEM (*n* = 3 independent experiments performed in duplicate).

We have reported previously on the specific binding of HS-731 to the three classical opioid receptors, MOR, DOR and KOR, in the rat brain and to the recombinant human receptors expressed in Chinese hamster ovary (CHO) cells (Table 1). HS-731 shows high binding affinities in the low nanomolar range to the MOR and DOR, and reduced affinity to the KOR [30,35]. In the present study, the first data on the binding affinity of HS-731 to the NOP receptor is reported. Competitive inhibition of [^3^H]nociceptin binding by HS-731 to the NOP receptor was assessed using in vitro competitive radioligand binding assays with membranes of CHO cells expressing the human NOP receptor. HS-731 displayed no substantial binding to the NOP receptor up to a concentration of 10 μM. In the same assay, the reference nociceptin ligand had a very high binding affinity (K_i_ = 0.17 ± 0.04 nM) to the NOP receptor (Figure 2 and Table 1).

### 2.2. Homology Modeling Is Suitable to Predict the Active State Human Nociceptin Receptor

**Figure 3:**
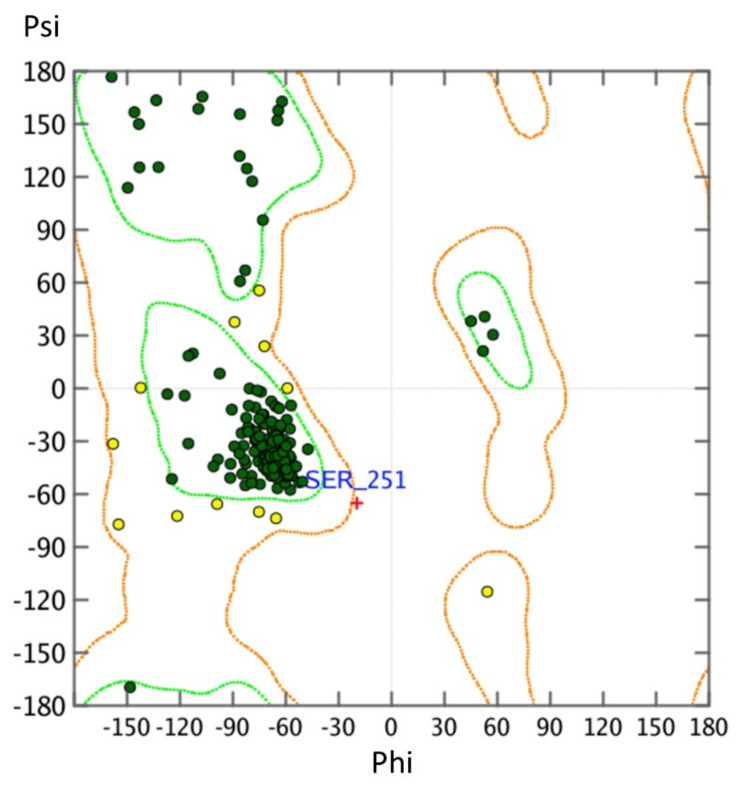
Ramachandran plot of the NOP receptor homology model. Angles within the orange range angles are plausible (yellow spheres) and angles within the green space are optimal (green spheres).

In order to characterize binding of HS-731 in a comprehensive way, an investigation of both the inactive conformation, but also the active conformation is necessary. Since no active-state crystal structure of the NOP receptor is available we modeled the active state human NOP receptor structure using the crystal structure of the κ-opioid receptor (KOR, PDB-ID: 6B73 [41]). Model generation was carried out as described in the Section 3 and resulted in a model with a 0.7 Å root mean square deviation (RMSD) between the α-carbon atoms between NOP receptor and active conformation KOR template, indicating a correct global fold. The NOP receptor homology model contains no atom clashes and only two phi/psi angle outliers, suggesting a high-quality homology model (Figure 3, see Section 3 for details).

### 2.3. Water Molecules Are Important for HS-731 Binding to the Opioid Receptors

Water-mediated hydrogen bonds between ligand and receptor are known to occur within opioid receptor crystal structures [41,42,43]. Both MOR and DOR x-ray crystal structures [42,43] contain crystal water molecules. In the MOR, three polar interactions between ligand and protein are mediated by water molecules, namely those to K233^5.39^, H297^6.52^ and Y148^3.33^ (the numbering refers to the mouse MOR; the respective residues in the human MOR are K235^5.39^, H299^6.52^ and Y150^3.33^, superscripts denote Ballesteros-Weinstein numbering [44]) were reported [42]. Mutagenesis studies have revealed all three residues to be involved in MOR binding and selectivity [45]. Therefore, the water molecules in the MOR structure were retained. The DOR structure with PDB-ID 6PT2 published by Claff et al. [43] contains three water molecules, which mediate interactions to K214^5.39^ and Y129^3.33^. In mutagenesis studies, Y129^3.33^ was shown to contribute to affinity and activity of DOR agonists [43,46], while K214^5.39^ contributes to agonist binding and selectivity [47]. Therefore, all three water molecules were retained. For the KOR, no crystal waters are experimentally resolved. ‘Interaction potential maps’ implemented in MOE were therefore used to identify a single potential conserved water position. The same workflow was applied to the NOP receptor homology model and the NOP receptor inactive crystal structure. For all three structures water molecule between the transmembrane helices 5 and 6 (TM5/TM6) were identified that are capable to mediate interactions to the backbone carbonyl of K5.39, an interaction highlighted previously in opioid receptors [41,42,43]. The predicted water molecule in the KOR occupies the same coordinates as a preserved water molecule present in the MOR (PDB-ID: 5C1M).

### 2.4. Docking Reveals a Common Binding Mode for HS-731 to the Opioid Receptors

**Figure 4:**
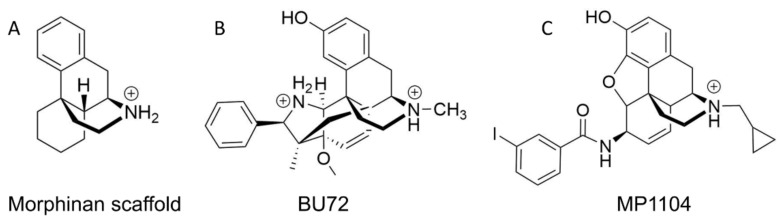
Chemical structures of (**A**) a morphinan scaffold and co-crystallized ligands (**B**) BU72 in MOR (PDB-ID: 5C1M) and (**C**) MP1104 in KOR (PDB-ID: 6B73) under physiological pH (7.4).

HS-731 contains the same morphinan scaffold (Figure 4) as the co-crystallized ligands of the active X-ray crystal structures used in this study (KOR co-crystalized with MP1104, PDB-ID: 6B73 [41], and MOR co-crystalized with BU72, PDB-ID: 5C1M [42], Figure 4). In contrast, the active state DOR structure used in this study contains a peptidic ligand [43]. Nevertheless, MP1104 is known to be a potent agonist at the MOR, DOR and KOR [41,48]. Thus, a maximal scaffold overlay of HS-731 and MP1104 or BU72, and additionally a common binding mode within the opioid receptors was aimed.

**Figure 5:**
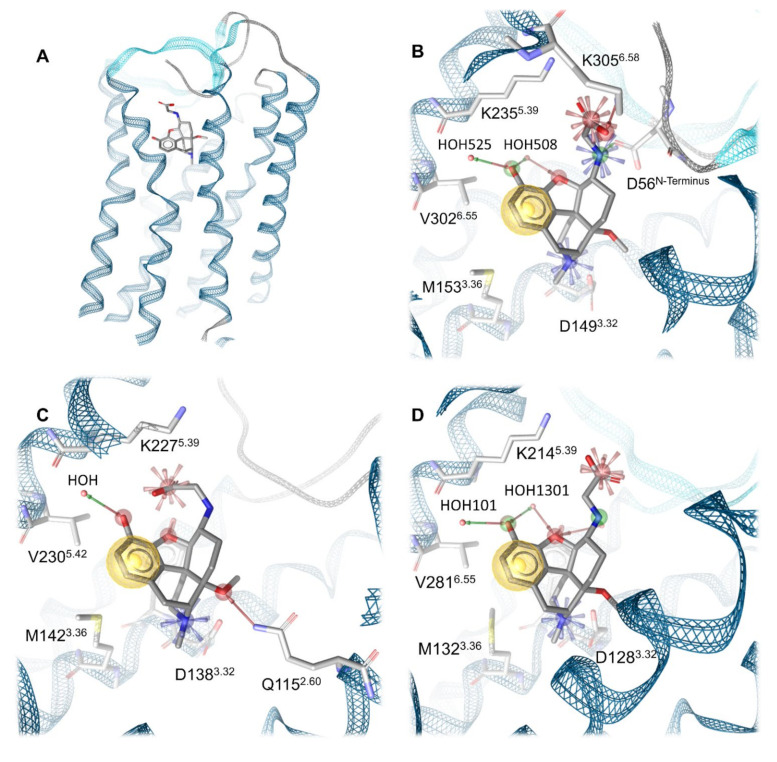
Binding modes of HS-731 to the classical opioid receptors. (**A**) Global view on the MOR binding pocket with docked HS-731. (**B**) Binding pocket of the MOR. Residues 297–303 and 322–325 are not shown for better visualization. (**C**) Binding pocket of the KOR. Residues 289–294 and 311–318 are not shown for better visualization. (**D**) Binding pocket of the DOR. Residues 275–282 are not shown for better visualization. Blue star indicates positive ionizable interactions, red stars negative ionizable interactions, yellow spheres lipophilic contacts, green arrow hydrogen bond donors and red arrows hydrogen bond acceptors. Water molecules are depicted as red spheres.

To obtain a common binding mode of HS-731 in all opioid receptors, we docked HS-731 into the prepared MOR, KOR, DOR x-ray crystal structures, as well as into the NOP receptor active state homology model and the NOP receptor inactive crystal structure. All protein structures contain water molecules in the TM5-6 region that is surmised to be important for ligand binding [41]. Docking revealed a common binding pose of HS-731 in the classical opioid receptors with the phenolic moiety establishing hydrogen bonding to the water molecules coordinating the K5.39 backbone carbonyl in TM5. The morphinan amine interacts with D3.32 via a salt bridge. The HS-731 carboxylate moiety points upwards to the extracellular domain (Figure 5A). An ionic interaction between the side chain of K5.39 and the carboxylate of HS-731 occurs in all the three classical opioid receptors. In the MOR, the carboxylate moiety also forms an ionic interaction with a second lysine positioned in TM6 (K305^6.58^). While K5.39 is conserved among the classical opioid receptors, residue 6.58 is not conserved, with the positively charged K305^6.58^ in the MOR, neutral W284^6.58^ in the DOR, and negatively charged E297^6.58^ in the KOR. Thus, HS-731 is only able to form ionic interactions with both lysine residues in the MOR, explaining the highest affinity of HS-731 to this receptor. In contrast, HS-731 only can exhibit one ionic interaction to K5.39 in the KOR and DOR (Figure 5B–C).

Even though residue 6.58 did not participate in an interaction with HS-731 in the KOR, it could have an influence on ligand binding. In the KOR, K227^5.39^ and E297^6.58^ could interact with each other in an ionic protein-protein-interaction. Subsequently the carboxylate of HS-731 would have to compete with E297^6.58^ for K227^5.39^ as interaction partner. This competition would likely weaken the strength of the ligand interaction to K227^5.39^ and reduces HS-731′s affinity to the KOR. The neutral W284^6.58^ in the DOR cannot participate in an ionic interaction. Nonetheless, it could take part in a weaker π-cation interaction with K214^5.39^. No geometrically plausible π-cation between W284^6.58^ and K214^5.39^ could be observed in our model. Subsequently, we surmise that W284^6.58^ does not influence ligand binding resulting in a better affinity value compared to the KOR. Additionally the non-conserved residue 6.58 is a known selectivity-determinant at the classical opioid receptors [45,47,49], and therefore its influence on ligand binding could contribute to the affinity pattern of HS-731 at the opioid receptors.

A feature only visible in the binding hypothesis generated at the MOR is an ionic interaction between the secondary amine of HS-731 and D56 of the N-terminus. The MOR is currently the only solved opioid receptor crystal structure in which the N-terminus covers the binding site [42]. Thus, possible interactions between HS-731 and the N-terminus of the KOR or DOR were not detectable, even though the unresolved parts of the N-termini of both receptors contain negatively charged residues that could be oriented towards the binding pocket. Hence, the ionic interaction between the secondary amine of HS-731 and the N-terminus of the MOR was not investigated in this study.

**Figure 6:**
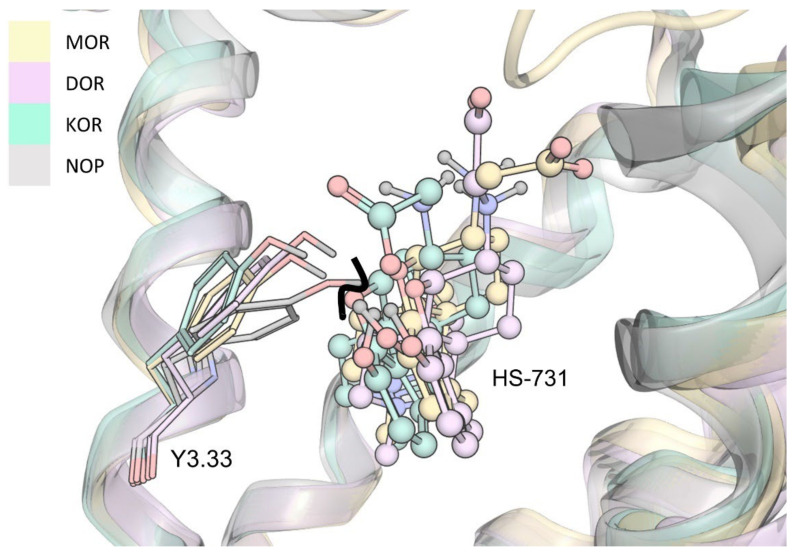
Superimposition of the NOP receptor and the classical opioid receptors in complex with HS-731. Atom clash is indicated by the bold black line.

Experimentally, HS-731 did not exhibit specific binding to the NOP receptor (Figure 2 and Table 1). Therefore, the generated binding hypothesis to the NOP receptor predominately served to give insights into the reasons for the lack of affinity to this receptor and to assess if HS-731 could be active in higher concentrations than experimentally tested. As there is no data about the activity profile of HS-731 at the NOP receptor available we conducted docking to the modeled active state NOP receptor as well as to the inactive state NOP receptor (crystal structure, PDB-ID: 5DGH). For the active state homology model no valid and plausible docking solution for the orthosteric binding pocket with the essential ionic interaction to D130^3.32^ could be obtained. Residue Y130^3.33^ is likely to cause this exclusion effect as it points deeper into the NOP receptor binding pocket than in the classical opioid receptors (Figure 6). A superimposition of NOP receptor with the classical opioid receptors in complex with HS-731 revealed atom clashes between the morphinan scaffold and Y130^3.33^ (Figure 6). This steric hindrance precludes HS-731 from binding to the active state NOP receptor orthosteric binding pocket. Additionally, Akuzawa et al. [50] demonstrated abolished binding of the endogenous ligand nociceptin to the NOP receptor mutant Q280A, which indicates an important role of Q280 in anchoring NOP agonists. Residue Q280 is positioned deep in the orthosteric binding pocket; therefore, it could not mediate HS-731 binding to the active conformation of the NOP receptor. Also, for the inactive state NOP receptor (as obtained from the crystal structure with PDB-ID 5DGH), no reasonable binding mode could be obtained. The binding site in the inactive NOP receptor conformation is enlarged allowing HS-731 to bind to the lower part of the orthosteric binding pocket as does the co-crystallized antagonist C-35. However, HS-731 adopted a different orientation within the binding pocket and exhibited a distinct interaction pattern compared to known NOP antagonists [51,52] as no 3D pharmacophore overlay could be detected (Figure S2). Furthermore, HS-731 was not able to stabilize its two charged moieties outside the morphinan scaffold in ionic interactions resulting in an enthalpically unfavorable binding mode. Unlike endorphins, enkephalins and dynorphins, the endogenous NOP receptor ligand nociceptin contains FGGF instead of YGGF in its message domain [50,51,53]. The additional hydroxyl group is considered to function as a discriminator feature between classical opioid receptors and the NOP receptor [51] with dynorphin A (Y^1^) showing no activity at the NOP receptor [54]. The phenyl group of nociceptin is considered to point deeply into the orthosteric binding pocket [51]. The discriminative hydroxyl group of HS-731 was similarly oriented further indicating an implausible binding mode for HS-731.

Altogether, the absence of affinity of HS-731 to the NOP receptor is in line with reports indicating that NOP ligands often exhibit binding and activity patterns to the NOP receptor not observed in the classical opioid receptors [1]. Furthermore, the lack of plausible docking poses implies inactivity of HS-731 to the NOP receptor even for high ligand concentrations. Hence, the binding poses at the NOP receptor were not further assessed in MD simulations.

### 2.5. Molecular Dynamics Simulations Revealed Additional Interactions for HS-731 Binding to the Opioid Receptors

To obtain dynamic information for the opioid receptor-HS-731 complexes, we performed MD simulations and analyzed the interactions using the in-house developed Dynophore software [55], that calculates dynamic pharmacophores (‘dynophores’). Table 2 shows the frequency of the ionic interactions between HS-731 and the three opioid receptors, MOR, DOR and KOR, during the simulations performed. Notably, the salt bridge between the morphinan amine and D3.32 that is known to be crucial for binding of positively charged ligands [56,57] occurred in 100% of the trajectory. In the case of the MOR, MD simulations resulted in the same four ionic interactions observed in the static model. The ionic interactions occurred with high frequencies, suggesting strong salt bridges between HS-731 and the MOR binding pocket (Table 2). Dynophore analysis obtained from the DOR and KOR complexes with HS-731 revealed additional, ionic interactions between the ligand and extracellular loops (DOR: R291^ECL3^; KOR: K200^ECL2^) that were not seen in the static model. The occurrence of ionic interactions with residues of the ECLs could be explained by a tilt of the loops towards the binding pocket during the simulations. Moreover, dynophore analysis revealed four stabilizing ionic interactions between ligand and protein in case of the KOR, but only three in case of the DOR (Table 2). Furthermore, the frequency of the ionic interaction between the morphinan amine and K5.39 is as frequent in the KOR as in the DOR, even though a lower frequency in case of the KOR was predicted due to possible intramolecular interaction between K227^5.39^ and E297^6.58^ as discussed in Section 2.4. The last two findings seem in disagreement with the higher affinity of HS-731 towards the DOR than to the KOR (Table 1). To explain these observations, we analyzed the geometry of the stabilizing salt bridges between HS-731 and the opioid receptors residues (Table 2) as described in the next section.

Detailed root-mean-square deviation (RMSD) plots of HS-731 and the protein backbone can be found in the Supplementary Materials (Figure S3, Figure S4, Figure S5, Figure S6, Figure S7 and Figure S8). Additionally, the supportive information provide a comparison of the binding modes of HS-731 at the end of the simulation time with the docking pose (Figure S9, Figure S10, and Figure S11).

### 2.6. Interaction Distance Assessment Confirms Binding Hypothesis

**Figure 7:**
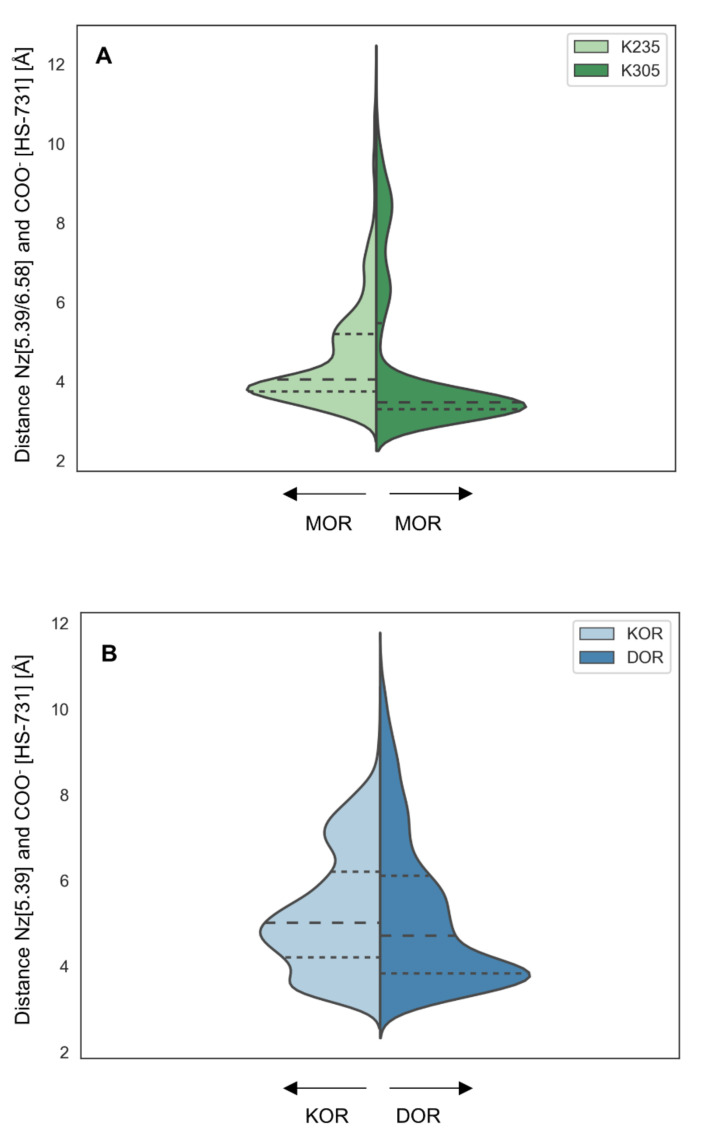
Ionic interaction distances. (**A**) Distances between K235^5.39^ (Nz) or K305^6.58^ (Nz) in the MOR and the carboxylate of HS-731. (**B**) Distances between K227^5.39^ (KOR, Nz) or K214^5.39^ (DOR, Nz) and the carboxylate moiety of HS-731. Dashed lines represent quantile. The width of the plot corresponds to the frequency of the measured distance.

We measured the distances between the interaction partner atoms to examine the quality of the ionic interactions occurring during MD simulations. Ionic interactions are known to be strongly distance-dependent and the energy of ionic interactions is determined by an exponential term, i.e., the strength of the interaction decreases rapidly by increasing distance [58]. The distance measurement between the carboxylate moiety of HS-731 and K5.39 at the MOR revealed short distances throughout the majority of the MD simulation (Figure 7A). The large extent of strong interactions implies stable ligand binding over the simulation time and the higher amount compared to the other opioid receptors contributes to the superior affinity of around one order of magnitude exhibited at the MOR.

The corresponding distance assessment at the DOR and KOR revealed far more short-distance interactions at the DOR than at the KOR (Figure 7B). Thus, even though the interaction frequency at the DOR and KOR was very similar, the interaction was much stronger at the DOR, explaining the increased affinity of HS-731 at the DOR compared to the KOR (Table 1). Additional interactions between the carboxylate and the basic residues in the ECLs in both receptors (R291^ECL3^ in the DOR, K200^ECL2^ in the KOR) only occurred with low frequency and long interaction distances rendering their effect on ligand binding negligible (Figure S12A). The ionic interaction in the KOR between E209^ECL2^ and the secondary amine of HS-731 also only occurred with low frequency and again the distance assessment revealed mostly long distances, rendering its effect on ligand binding trivial (Figure S12B).

**Figure 8:**
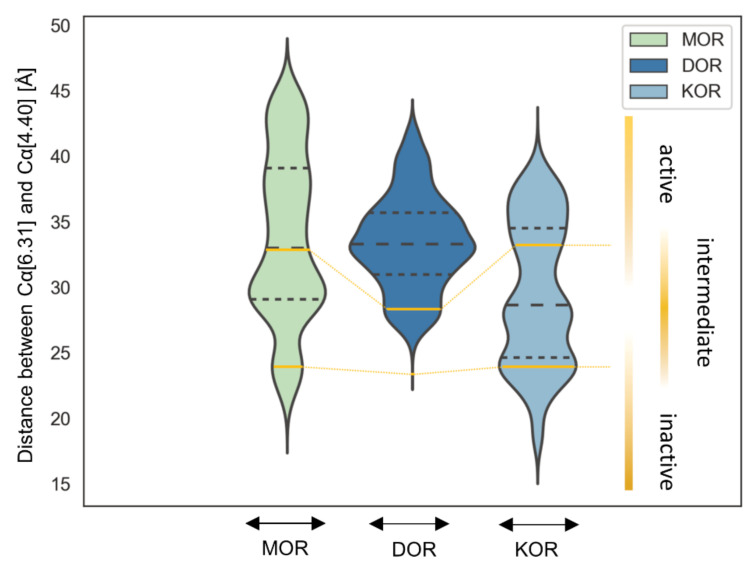
TM6 translocation. Measurement between the alpha carbon atoms of 6.31 at the bottom of TM6 (MOR: R278^6.31^, DOR: R257^6.31^, KOR: R270^6.31^) and 4.40 at the bottom of TM4 (MOR: R184^4.40^, DOR: A163^4.40^, KOR: L173^4.40^) over the simulation time. The width of the plot corresponds to the frequency of the measured distance. Dashed black lines represent quantile. Yellow solid lines indicate the analog measured distances at the active state crystal structures (PDB-ID: 5C1M for MOR, 6PT2 for DOR and 6B73 for KOR) and inactive state crystal structures (PDB-ID: 4DKL for MOR, 4N6H for DOR and 4DJH for KOR).

To explain the activity profile of HS-731 as a partial agonist at the KOR (Table 1), the possible interaction between K227^5.39^ and E297^6.58^ to the KOR was assessed. This is because a salt bridge between K227^5.39^ and E297^6.58^ at the KOR is assumed to hamper KOR activation in that the interaction between the TM5 and TM6 hinders TM6 from its outward movement [59]. At the same time, the translocation of TM6 is important for receptor activation at GPCRs like the opioid receptors [4,60] and interactions between TM5 and TM6 are considered to hamper activation in other GPCRs [61]. This hypothesis is supported by the fact that the salt bridge between K227^5.39^ and E297^6.58^ at the KOR only occurs in the inactive conformation (PDB-ID: 4DJH [56]), but was broken up in the active crystal structure (PDB ID: 6B73 [41]). The partial adoption of an intermediate state conformation with a less pronounced outward movement due to K227^5.39^–E297^6.58^ interaction would explain the partial agonism of HS-731 at the KOR. Our simulation shows that the two residues interact with each other during 45.6% of the time indicating that the surmised intermediate state is indeed relevant. Furthermore, the proposed hindered TM6 outward movement at KOR was confirmed by a distance measurement between the alpha carbonyl atoms of the opposing residues 6.31 at the bottom of TM6 (MOR: R278^6.31^, DOR: R257^6.31^, KOR: R270^6.31^) and 4.40 at the bottom of TM4 (MOR: R184^4.40^, DOR: A163^4.40^, KOR: L173^4.40^) over the simulation time (Figure 8). Thus, the K227^5.39^–E297^6.58^ interaction appears to induce a less active conformation at KOR explaining the observed partial agonism of HS-731 at the KOR. A comparison between the active state KOR (PDB-ID: 6B73) and one exemplary intermediate state conformation can be found in the Supplementary Materials (Figure S13).

In the case of the MOR and DOR, which do not exhibit negatively charged residues in the upper half of TM6, no similar interaction occurred, in accordance with the HS-731 full agonism observed at these receptors (Table 1). To ensure that all influencing factors for TM5–TM6 interactions in the DOR were properly considered, the occurrence of cation–π-interactions between W284^6.58^ and K214^5.39^ were determined. As surmised this interaction was not detectable in MD simulations confirming the hypothesis of partial agonism in the presence of TM5–TM6 interactions.

Definition of the intermediate state for all opioid receptors based on the TM6 deflection measured between the alpha carbonyl atoms of the opposing residues 6.31 and 4.40 at the respective active state crystal structures (PDB-ID: 5C1M for MOR, 6PT2 for DOR and 6B73 for KOR) and inactive state crystal structures (PDB-ID: 4DKL for MOR, 4N6H for DOR and 4DJH for KOR) clearly indicates a maximum within the intermediate area for KOR, but also for MOR while DOR only very rarely adopts such a state (Figure 8). Nonetheless, the number of intermediate state conformations observed for all three opioid receptors during the simulation time reflects the order of activation potential measured in the [^35^S]GTPγS binding assay (Table 1). The KOR-HS-731 complex exhibits 51.9% of the time an intermediate state conformation corresponding to 82% stimulation in the [^35^S]GTPγS binding assay and partial agonism. The MOR-HS-731 complex in contrast only adopts an intermediate conformation in 44.6% of the simulation time correlating to 98% stimulation in the [^35^S]GTPγS binding assay and full agonism. The DOR-HS-731 complex reaches 103% stimulation in the [^35^S]GTPγS binding assay and full agonism with only 5.3% intermediate states.

## 3. Materials and Methods

### 3.1. Chemicals and Materials

HS-731 was prepared as previously described [29]. Radioligand [^3^H]nociceptin (119.4 Ci/mmol) was purchased from PerkinElmer (Boston, MA, USA). Tris(hydroxymethyl) aminomethane (Tris), bovine serum albumin (BSA) polyethylenimine (PEI) and nociceptin were obtained from Sigma-Aldrich Chemicals (St. Louis, MO, USA). Cell culture media and supplements were obtained from Sigma-Aldrich Chemicals (St. Louis, MO, USA). All other chemicals were of analytical grade and obtained from standard commercial sources. Test compounds were prepared as 1 mM stocks in water and further diluted to working concentrations in 50 mM Tris-HCl buffer (pH 7.4).

### 3.2. Cell Culture and Membrane Preparation

CHO cells stably expressing the human NOP receptor (CHO-hNOP cell line) was kindly provided by Dr. Lawrence Toll (SRI International, Menlo Park, CA, USA). CHO-hNOP cells were grown at 37 °C in Dulbecco’s Modified Eagle’s Medium (DMEM) culture medium supplemented with 10% fetal bovine serum (FBS), 0.1% penicillin/streptomycin, 2 mM L-glutamine and 0.4 mg/mL geneticin (G418). Cells were maintained in a humidified atmosphere of 95% air and 5% CO_2_. Membranes from CHO-hNOP cells were prepared as described previously [62]. Briefly, CHO-hNOP cells grown at confluence were removed from the culture plates by scraping, homogenized in 50 mM Tris-HCl buffer (pH 7.7) using a Polytron homogenizer, then centrifuged once and washed by an additional centrifugation at 27,000× g for 15 min at 4 °C. The final pellet was resuspended in 50 mM Tris-HCl buffer (pH 7.7) and stored at –80 °C until use. Protein content of cell membrane preparations was determined by the method of Bradford using BSA as the standard [63].

### 3.3. [^3^H]NOP Receptor Binding Assay

Competitive binding assays at the human NOP receptor stably transfected into CHO cells were performed according to the published procedure [62]. Cell membranes (15 µg) were incubated in 50 mM Tris-HCl buffer (pH 7.4) with [^3^H]nociceptin (0.1 nM) and various concentrations of test compounds in a final volume of 1 mL, for 60 min at 25 °C. Non-specific binding was determined using 10 µM of unlabeled nociceptin. After incubation, reactions were terminated by rapid filtration through 0.5% PEI-soaked Whatman GF/C glass fiber filters. Filters were washed three times with 5 mL of ice-cold 50 mM Tris-HCl buffer (pH 7.4) using a Brandel M24R cell harvester (Brandel, Gaithersburg, MD, USA). Radioactivity retained on the filters was counted by liquid scintillation counting using a Beckman Coulter LS6500 (Beckman Coulter Inc., Fullerton, CA, USA). Inhibition constant (K_i_, nM) values were determined by the method of Cheng and Prusoff [64] from concentration-response curves by nonlinear regression analysis using the GraphPad Prism 5.0 Software (GraphPad Prism Software Inc., San Diego, CA, USA). All experiments were performed in duplicate and repeated three times with independently prepared samples. Data are presented as means ± SEM.

### 3.4. Protein Preparation and Modeling of the KOR Active Conformation

For classical opioid receptors, X-ray crystal structures of the active state proteins are published and provided in the protein data bank (PDB [65]). The respective structures with PDB-IDs 5C1M for the MOR [42], 6PT2 for the DOR [43] and 6B73 for the KOR [41] were prepared using MOE v2020.0901 [66]. The X-ray crystal structure of the inactive state NOP receptor (PDB-ID: 5DGH) was prepared analog. Only the chain with the best resolution was processed. Fusion proteins (antibody fragment in MOR, thermostabilized cytochrome b562 (BRIL) in the DOR, nanobody in the KOR) and the unresolved parts of the N-terminus, as well as of the C-terminus of the opioid receptors were deleted. Thermostabilizing mutations in the DOR and KOR were subsequently reverted to the human wild-type sequence obtained from the UniProt-Databank [67] (human DOR: P41143, human KOR: P41145). The MOR structure (PDB-ID: 5C1M) is of a murine receptor. Hence, the sequence was manually mutated to obtain the human wild-type MOR model (UniProt-ID: P35372). The NOP receptor structure already contained the human sequence. Missing side chain atoms were automatically generated using the protein builder integrated in MOE. The unresolved parts of ECL2, ECL3 and ICL3 of the KOR and ICL2 of the NOP receptor were modeled using the loop modeler panel within MOE. To obtain high quality structures, Ramachandran outliers [68] and atom clashes were resolved using energy minimization with the OPLS-AA force field [69].

Homology modeling of the active state NOP receptor was performed using MOE v2020.0901 with default settings in a similar as described in [70]. The chain with the best resolution (3.10 Å) of the active KOR structure (PDB-ID: 6B73, sequence identity of 59% and sequence similarity of 73%) with the NOP receptor (Figure S1) served as a template. The protein target sequence (human NOP receptor) was obtained from the UniProt-Database (human NOP receptor P41146). Both Ramachandran outliers as shown in Figure 3 are located in flexible loops far away from the binding site (T206 of extracellular loop 2, ECL2, and S251 of intracellular loop 3, ICL3). Hence, we assume that these Ramachandran outliers are unlikely to influence ligand binding. Visual inspection revealed that the side chain orientations of the residues forming the orthosteric binding pocket, including D3.32 (number denote Ballesteros–Weinstein numbering [44]), responsible for the crucial ionic interaction between opioids and their receptors, show a similar orientation in the generated model as in the template.

‘Interaction potential maps’ as implemented in MOE v2020.0901 were used to determine putatively relevant water molecules inside the binding site of the KOR (resolution too low to determine co-crystallized waters) and the NOP receptor (homology model without water coordinates; too low resolution in the crystal structure). The interaction potential is an energy-based function that probes water molecules within the protein and calculates the interaction energy between water molecule and protein [66]. For this calculation the KOR binding site was defined as all residues within 4.5 Å around the crystalized ligand MP1104 in the KOR structure (PDB-ID: 6B73). Since the KOR and NOP receptor share a high sequence identity (59%) the same resides were used to define the NOP binding site in the active state homology model. For the NOP receptor crystal structure again, all residues within 4.5 Å around the crystalized ligand C-35 were used.

### 3.5. Protein-Ligand Docking

The starting conformation of HS-731 (IUPAC name: 2-[(4,5α-epoxy-3-hydroxy-14β-methoxy-17-methylmorphinan-6β-yl)amino]acetic acid) was generated using Corina v3.00 [71,72]. All five opioid receptor structures were protonated at a pH of 7.0 using the protonate 3D function [73] included in MOE (v2020.0901). GOLD v5.2 [74] was used for docking HS-731 into the receptors. The binding site was defined as a 20 Å sphere around the side chain carboxylate C (γC)-atom of D3.32 and restricted to the solvent-accessible surface. Pyramidal nitrogen atoms in the ligand were allowed to flip during the docking process. A total of 30 genetic algorithm runs per receptor structure were performed, generating diverse solutions (the root mean square deviation between docking poses was more than 1.5 Å). The generated binding hypotheses were scored using the GoldScore docking function [75,76]. The search efficiency was held at 100%. A constraint maintaining a maximum distance of 5.5 Å between the nitrogen in the morphinan scaffold and the γC-atom of D3.32 was set to ensure a crucial ionic interaction [41,56,57,77].

The obtained binding poses were energy-minimized in the protein environment using the MMFF94 force field [78] implemented in LigandScout v4.4.3 [79,80]. The binding poses of HS-731 in complex with the MOR, DOR and KOR were visually inspected and filtered according to the reported binding mode of the morphinan scaffold of opioid agonist BU72 co-crystallized with the MOR (PDB-ID: 5C1M [42]) and the morphinan scaffold of the opioid agonist MP1104 co-crystallized with the KOR (PDB-ID: 6B73 [41]). Additionally, MP1104-KOR interactions were used to score the DOR docking results as MP1104 also exhibits full agonism at the DOR. The relevant interactions are summarized in Table 3. Rescoring of the MOR and KOR clearly identified one docking result as most plausible that was chosen for further evaluation. At the DOR however, several docking results were scored equal. Thus, the pose with the lowest distance between the positively charged nitrogen in the morphinan scaffold and the carboxylate of D3.32 out of the best scored docking results was chosen at the DOR.

None of the crystallized opioid ligands exhibit agonist activity to the NOP receptor, but due to high identity and similarity to the classical opioid receptors (Figure S1) a similar binding mode of HS-731 in all active state opioid receptors was assumed. MP1104 shares the morphinan scaffold of HS-731 and an alignment and superposition of the KOR crystal structure and the NOP receptor homology model revealed the same orientation of the residues that interact with MP1104 in the MP1104-KOR-complex and their NOP receptor equivalent, with the exception of Y131^3.33^. Therefore, the binding poses were evaluated according to the geometry of the other interactions detected in the MP1104-KOR complex (Table 3). For the inactive state NOP receptor (PDB-ID: 5DGH) the orientation and interaction pattern of the cocrystallized ligand C-35 was used to evaluate the docking poses. C-35 only exhibit the crucial ionic interaction towards D130^3.32^ as well as several hydrophobic interactions (to I127^3.29^, I129^3.31^, Y131^3.33^, M134^3.36^, V279^6.51^, V283^6.55^).

### 3.6. Molecular Dynamics Simulations and Analysis to Evaluate Docking Poses

Five replicates of molecular dynamics (MD) simulations of 100 ns were performed for each receptor-ligand complex. The systems were set up using Maestro v2020-4 [81] and parametrized using the OPLS 2005 force field [82,83]. The MD simulations were performed using Desmond v2020-4 [84]. The protein was placed in a cubic box with 10 Å padding either side to the protein surface filled with TIP4P water molecules [85] and ions (0.15 M NaCl), to ensure isotonic conditions, and was embedded in a 1-palmitoyl-2-oleoylphosphatidylcholine (POPC) bilayer. The membrane placement was carried out according the OPM database (PDB-ID: 5C1M for the MOR, 6PT2 for the DOR, 6B73 for the KOR). The simulation was performed under periodic boundary conditions as an NPγT ensemble, i.e., a constant number of particles, pressure (1.01325 bar), lateral surface tension (0 N/m) and temperature (300 K) throughout the simulation. Each simulation resulted in 1000 system conformations, according to a 100 ps recording interval. VMD v1.9.3 [86] was used to center the protein and to align the trajectory onto the backbone heavy atoms of the starting protein conformation.

For MD simulation analysis, dynamic pharmacophores, so called dynophores [55,87], were calculated. Dynamic pharmacophores encompass pharmacophoric information derived from an ensemble of protein conformations obtained from MD simulations. Interactions are grouped into feature groups according to their interaction type (e.g., lipophilic interaction, hydrogen bond acceptor, hydrogen bond donator). The interaction occurrence over the trajectory of each interaction group is statistically determined and reported as percentages. The dynophore algorithm is implemented the ilib framework, on which also LigandScout [79,80] is based upon. To assess the quality of interactions occurring during the MD simulations distances between HS-731-COO-(C-atom)-KOR-K227^5.39^ (Nz), HS-731-COO-(C-atom)-DOR-K214^5.39^ (Nz), HS-731-COO-(C-atom)-MOR-K235^5.39^ (Nz) and HS-731-COO-(C-atom)-MOR-K305^6.58^ (Nz), HS-731-COO-(C-atom)–DOR-R291 (Cz), HS-731-COO-(C-atom)-KOR-K200, and HS-731-secundary amine-KOR-E209 (CD) were measured using VMD. The violin plots (Figure 7) representing the distribution of measured distances were generated using the python v3.8.5 [88] packages seaborn v0.11.2 [89] and matplotlib v3.4.3 [90].

## 4. Conclusions

In this study, we assessed the difference in binding affinity and activity values of the peripheral opioid antinociceptive, HS-731, at the opioid receptors, and generated a binding hypothesis at each opioid receptor subtype. HS-731 shows extensive ionic interactions with the classical opioid receptors, MOR, DOR and KOR, and the differences in the frequency and quality of those interactions mediate differences in the affinity and activity of HS-731 to these receptors. At the MOR, HS-731 forms four ionic interactions over the majority of the MD simulations. At the DOR and KOR, there were only two noteworthy ionic interactions present. A closer examination of the interaction quality facilitated by an interaction distance assessment revealed by far the strongest ionic interactions at the MOR followed by the DOR. The quality at the KOR was much weaker than at the DOR. A salt bridge between K227^5.39^ and E297^6.58^ was observed in about 50% in the case of the KOR. This interaction is likely to cause the KOR to adopt an intermediate-state conformation as supported by the decreased distance between the bottom of TM6 and TM4 as a surrogate parameter for the TM6 translocation and GPCR activation, and therefore could explain the partial agonism of HS-731 to the KOR. The MOR and DOR that did not exhibit TM5-TM6 ionic interactions, and thus were not forced to adopt an intermediate state conformation are able to be fully activated by the agonist HS-731.The present results highlight the importance of ionic interactions for the binding of the 6β-glycine substituted agonist HS-731 to the opioid receptors, and accentuate the non-conserved residue 6.58 and the N-terminus, as important selectivity determinants for the classical opioid receptors. We experimentally demonstrate that HS-731 displayed no substantial binding to the NOP receptor. We surmise that Y131^3.33^ is responsible for this observation, in that it points further into the active state binding pocket than in the classical opioid receptors and prevents HS-731 binding within the orthosteric binding pocket. Furthermore, the hydroxyl group of HS-731 is likely to abolish ligand binding to the NOP receptor in that it mimics the tyrosine within the message address of endogenous peptides for the classical opioid receptors instead of the phenylalanine within the message address of the NOP receptor agonist nociceptin.

In conclusion, our findings offer significant structural insights into HS-731 interactions with the opioid receptors that are important for understanding the pharmacology of this peripheral opioid analgesic.

## Figures and Tables

**Table 1:** In vitro binding affinities and agonist activities of HS-731 at the opioid receptors.

Receptor	Rat Opioid Receptor	Human Opioid Receptor		
	Binding affinity	Binding affinity	Functional activity	
	K_i_ (nM)	K_i_ (nM)	EC_50_ (nM)	% stim.
MOR	0.83 ± 0.02 ^a^	0.90 ± 0.14 ^b^	3.78 ± 0.73 ^b^	98 ± 9 ^b^
DOR	7.86 ± 0.64 ^a^	10.1 ± 2.7 ^b^	7.92 ± 1.63 ^b^	103 ± 7 ^b^
KOR	44.8 ± 0.1 ^a^	- ^c^	361 ± 154 ^b^	82 ± 9 ^b^
NOP	- ^c^	>10,000	- ^d^	- ^d^

^a^ Binding affinities (K_i_, nM) to the opioid receptors in the rat brain were determined in competitive radioligand binding assays; data from [30]. ^b^ Binding affinities (K_i_, nM) to the human opioid receptors expressed in CHO cells were determined in competitive radioligand binding assays; data from [35]. Potencies (EC_50_, nM) and efficacies (% stimulation expressed as percentage relative to the maximum effect of a selective, full opioid agonist) to the human opioid receptors expressed in CHO cells were determined in the [^35^S]GTPγS binding assays; data from [35]. ^c^—denotes not determined. ^d^—denotes not applicable. Values are means ± SEM (*n* = 3 independent experiments performed in duplicate).

**Table 2:** Ionic interaction occurrence between HS-731 and the three classical opioid receptors during MD simulations.

InteractionType	Interaction Partners			
	HS-731	MOR	DOR	KOR
Cationic interaction	morphinan amine	D149^3.32^(100%)	D128^3.32^ (100%)	D138^3.32^ (100%)
Cationic interaction	secondary amine	D56^N-terminus^ (73.7%)	Not present	E209^ECL2^ (12.5%)
Anionic interaction	Carboxylate	K235^5.39^ (81.3%)	K214^5.39^ (65.0%)	K227^5.39^ (63.3%)
		K305^6.58^ (75.0%)	R291^ECL3^ (9.2%)	K200^ECL2^ (15.7%)

The frequency is given as an average of five simulation replicates per system.

**Table 3:** Ligand–receptor interactions used for rescoring of docking results.

Interaction	BU72	MP1104		
	MOR	DOR	KOR	NOP
PI	D149^3.32^	D128^3.32^	D138^3.32^	D130^3.32^
HY	M153^3.36^	M132^3.36^	M142^3.36^	M134^3.36^
HY	V238^5.42^	V217^5.42^	V230^5.42^	I219^5.42^
HY	I298^6.51^	-	-	-
HY	V302^6.55^	V281^6.55^	I294^6.55^	283^6.55^
HY	W320^7.35^	-	-	-
HBA	-	Y129^3.33^	Y139^3.33^	-
HBA/HBD	HOH525/508	HOH101/1301/1302	HOH	HOH

PI, positive ionizable interaction; HY, hydrophobic interaction; HBA, hydrogen bond acceptor; HBD, hydrogen bond donator; HOH refers to water molecules.

## Data Availability

Data is available from the authors upon reasonable request.

## Supplementary Materials

The following are available online, Figure S1: Sequence identity and similarity among the opioid receptors, Figure S2: Docking pose of HS-731 to the inactive NOP receptor, Figure S3: Root mean square deviation of HS-731 in complex with the MOR over the simulation time, Figure S4: Root mean square deviation of the MOR backbone atoms in complex with HS-731 over the simulation time, Figure S5: Root mean square deviation of HS-731 in complex with the DOR over the simulation time., Figure S6: Root mean square deviation of the DOR backbone atoms in complex with HS-731 over the simulation time, Figure S7: Root mean square deviation of HS-731 in complex with the KOR over the simulation time, Figure S8: Root mean square deviation of the KOR backbone atoms in complex with HS-731 over the simulation time, Figure S9: Comparison of the binding modes of HS-731 at the MOR derived by docking and after MD simulations, Figure S10: Comparison of the binding modes of HS-731 at the DOR derived by docking and after MD simulations. Figure S11: Comparison of the binding modes of HS-731 at the KOR derived by docking and after MD simulations. Figure S12: Ionic interaction distances. Figure S13: Comparison between the active state KOR and the intermediate state KOR.

## Sample Availability

Sample of compound is available from the authors.
