# Supplementary material for: Mechanistic Characterization of the Pharmacological Profile of HS-731, a Peripherally Acting Opioid Analgesic, at the µ-, δ-, κ-Opioid and Nociceptin Receptors
Source: Molecules. 2022 Jan 28;27(3):919. doi: 10.3390/molecules27030919 (PMC8840597; doi:10.3390/molecules27030919)
Supplement: Supplementary file 1 [file molecules-27-00919-s001.zip › molecules-1510541-supplementary.pdf]

Supplementary Materials

Mechanistic characterization of the pharmacological profile of HS-731, a peripherally acting opioid analgesic, at the μ-, δ-, κ-opioid and nociceptin receptors

Kristina Puls [1], Helmut Schmidhammer [2], Gerhard Wolber [1,*] and Mariana Spetea [2,*]

[1] Department of Pharmaceutical Chemistry, Institute of Pharmacy, Freie Universität Berlin; Königin-Luise-Str. 2+4, D-14195 Berlin, Germany; kristina.puls@fu-berlin.de (K.P.), gerhard.wolber@fu-berlin.de (G.W.)

[2] Department of Pharmaceutical Chemistry, Institute of Pharmacy and Center for Molecular Biosciences Innsbruck (CMBI), University of Innsbruck, Innrain 80-82, 6020 Innsbruck, Austria; mariana.spetea@uibk.ac.at (M.S.); helmut.schmidhammer@uibk.ac.at (H.S.)

* Correspondence: gerhard.wolber@fu-berlin.de (G.W.), Tel.: +49 30 838 52686; mariana.spetea@uibk.ac.at (M.S.), Tel.: +43 512 507 58277

Identity

	1.	2.	3.	4.
1. hNOP		58.5	57.4	57.9
2. hKOR	59.1		67.9	66.7
3.hMOR	57.4	67.2		69.7
4.hDOR	58.1	66.2	69.9	

Similarity

	1.	2.	3.	4.
1. hNOP		72.6	71.3	71.4
2. hKOR	73.3		81.8	79.8
3.hMOR	71.3	80.9		79.8
4.hDOR	71.6	79.3	80.1	

Figure S1. Sequence identity and similarity among the opioid receptors (On the left identity, on the right similarity. Values were measured for the truncated sequences corresponding to residues 35 to 330 of the NOP receptor. The darker the box, the higher the identity or similarity.

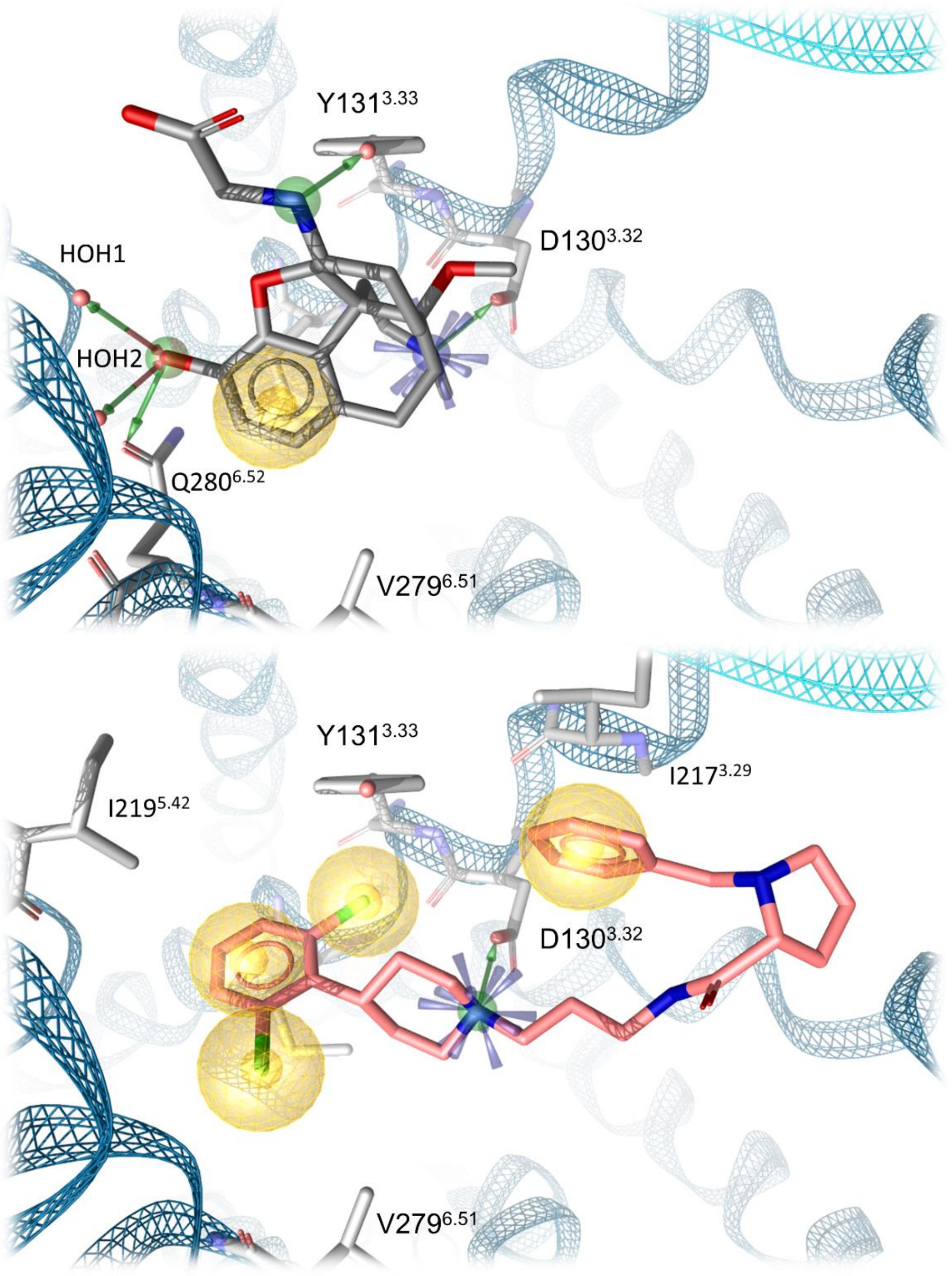

Figure S2: Docking of HS-731 to the inactive NOP receptor. Top: Implausible HS-731 confirmation from unsuccessful docking experiments in PDB-ID 5DGH explaining experimental results. Bottom: Co-crystallized antagonist C-35 from 5DGH illustrating a plausible binding mode for this compound class for comparison. Blue stars indicate positive charge interactions, yellow spheres lipophilic contacts, green arrow hydrogen bond donors and red arrows hydrogen bond acceptors. Water molecules are depicted as red spheres.

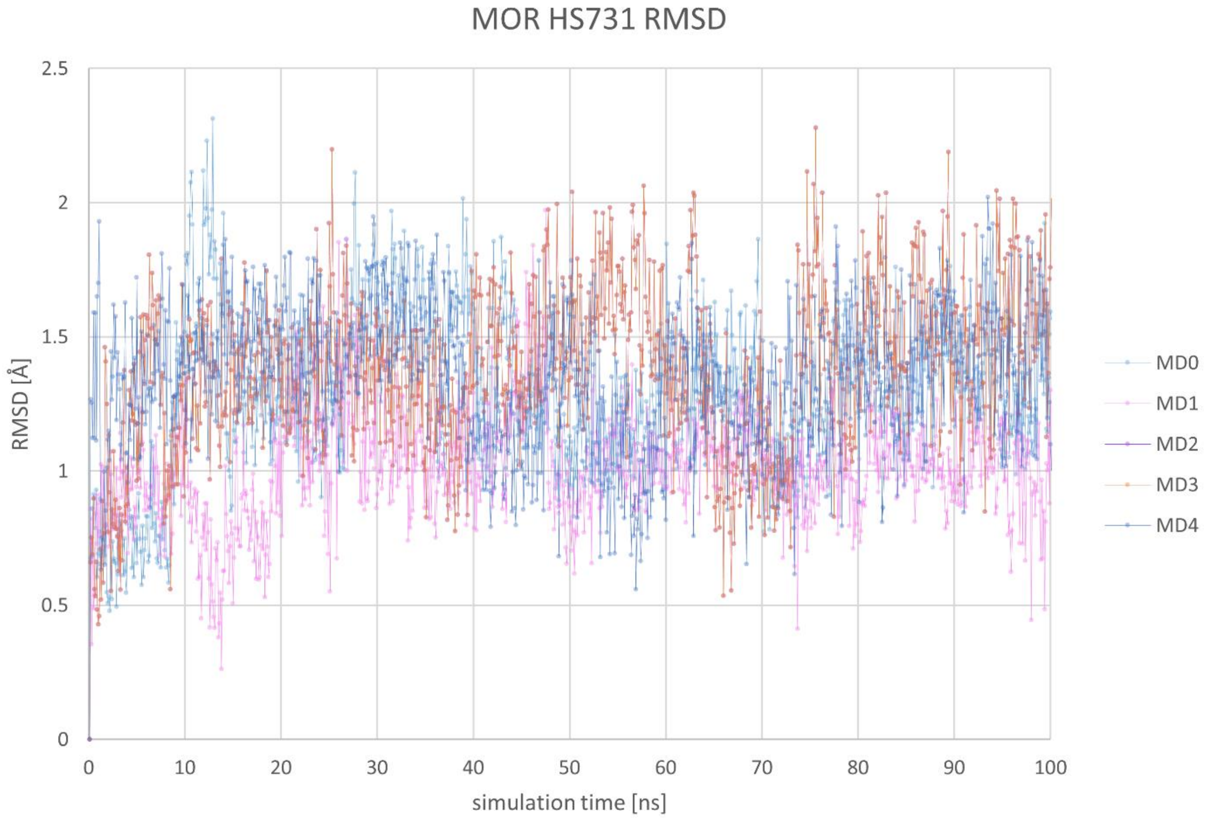

Figure S3. Root mean square deviation of HS-731 in complex with the MOR over the simulation time.

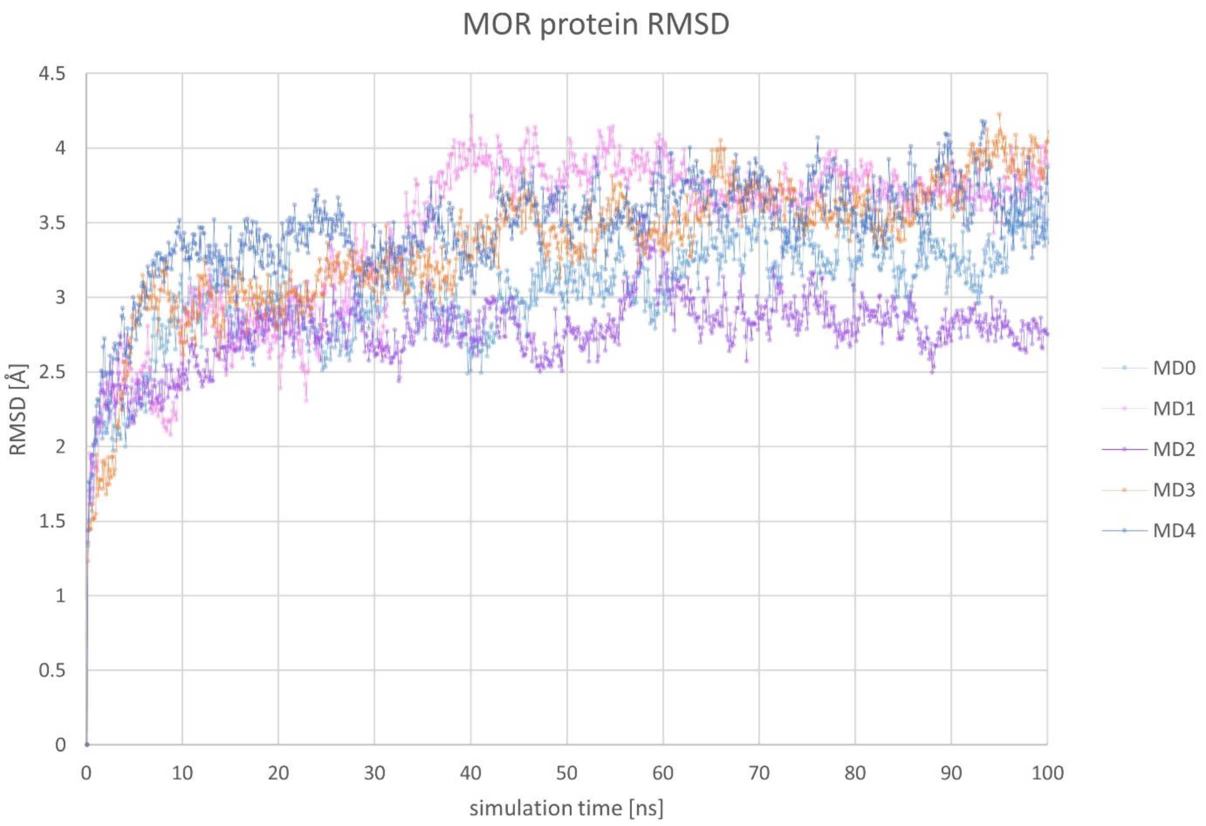

Figure S4. Root mean square deviation of the MOR backbone atoms in complex with HS-731 over the simulation time.

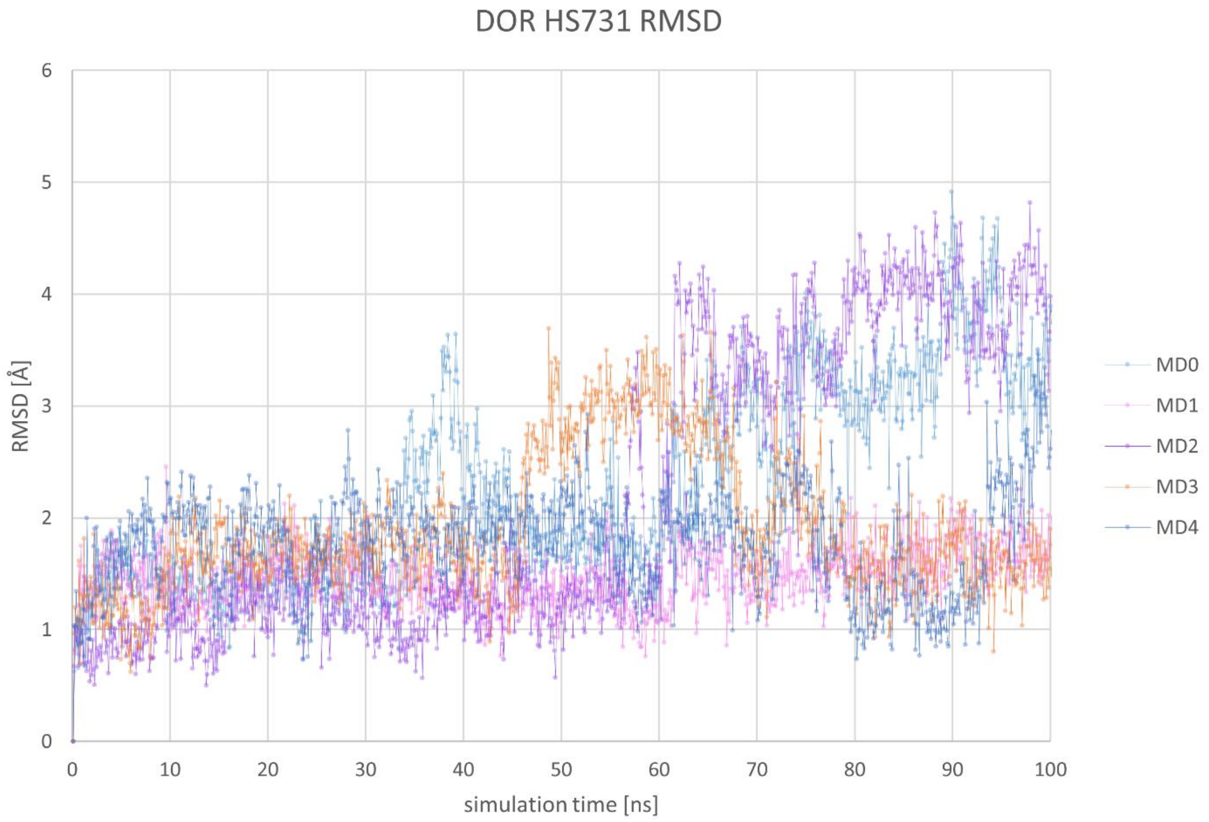

Figure S5. Root mean square deviation of HS-731 in complex with the DOR over the simulation time.

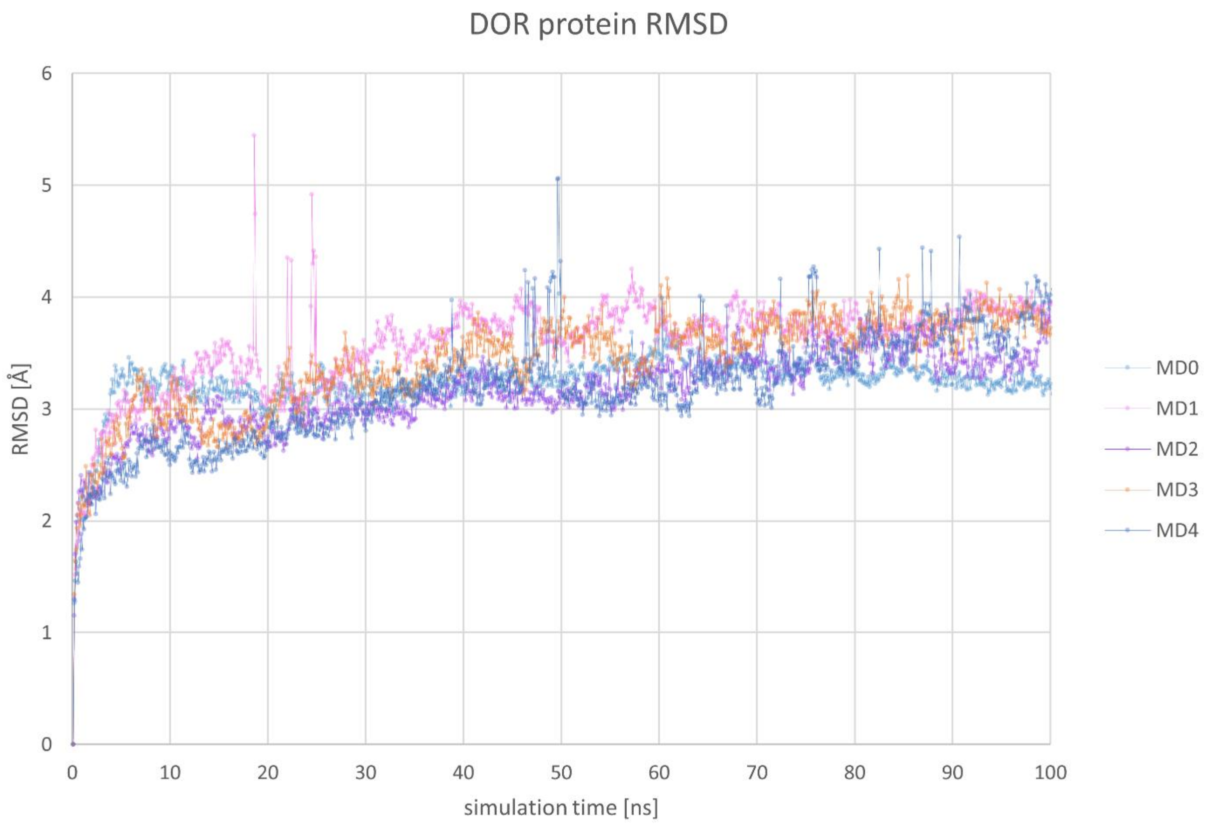

Figure S6. Root mean square deviation of the DOR backbone atoms in complex with HS-731 over the simulation time.

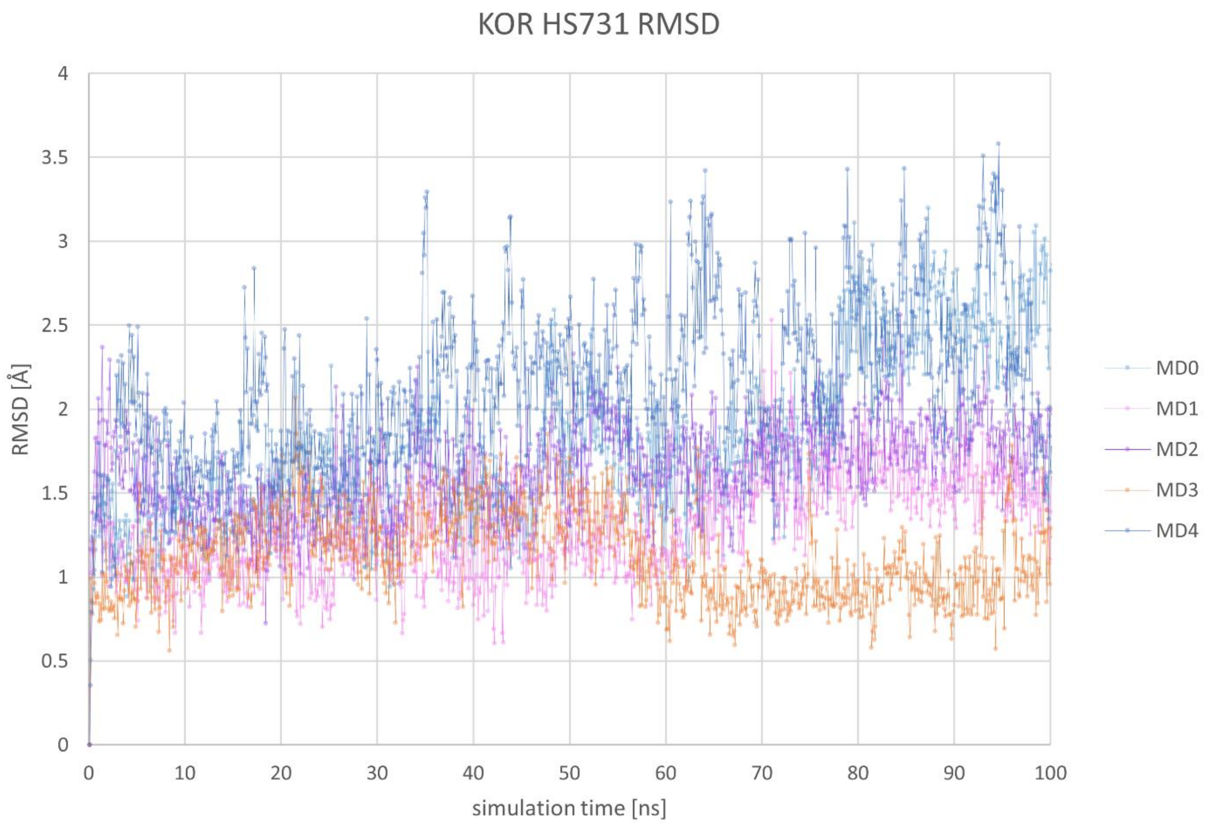

Figure S7. Root mean square deviation of HS-731 in complex with the KOR over the simulation time.

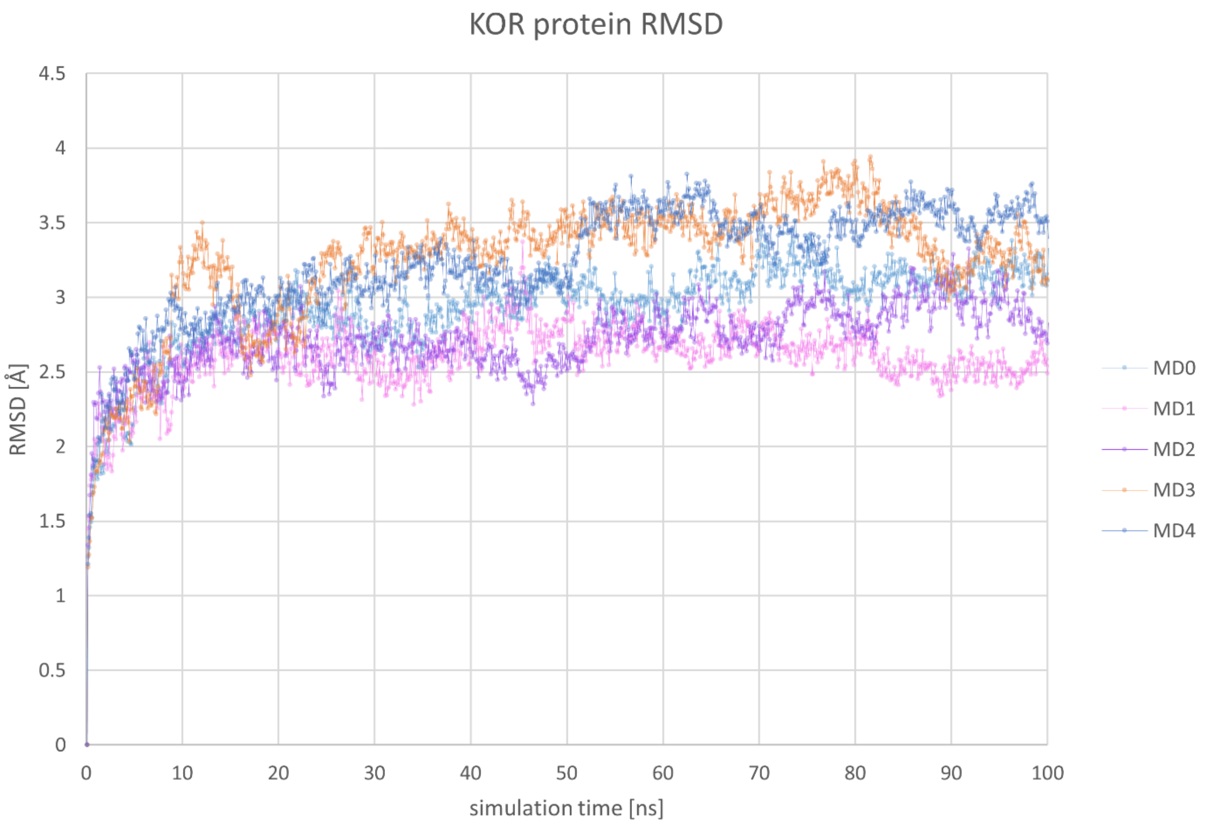

Figure S8. Root mean square deviation of the KOR backbone atoms in complex with HS-731 over the simulation time.

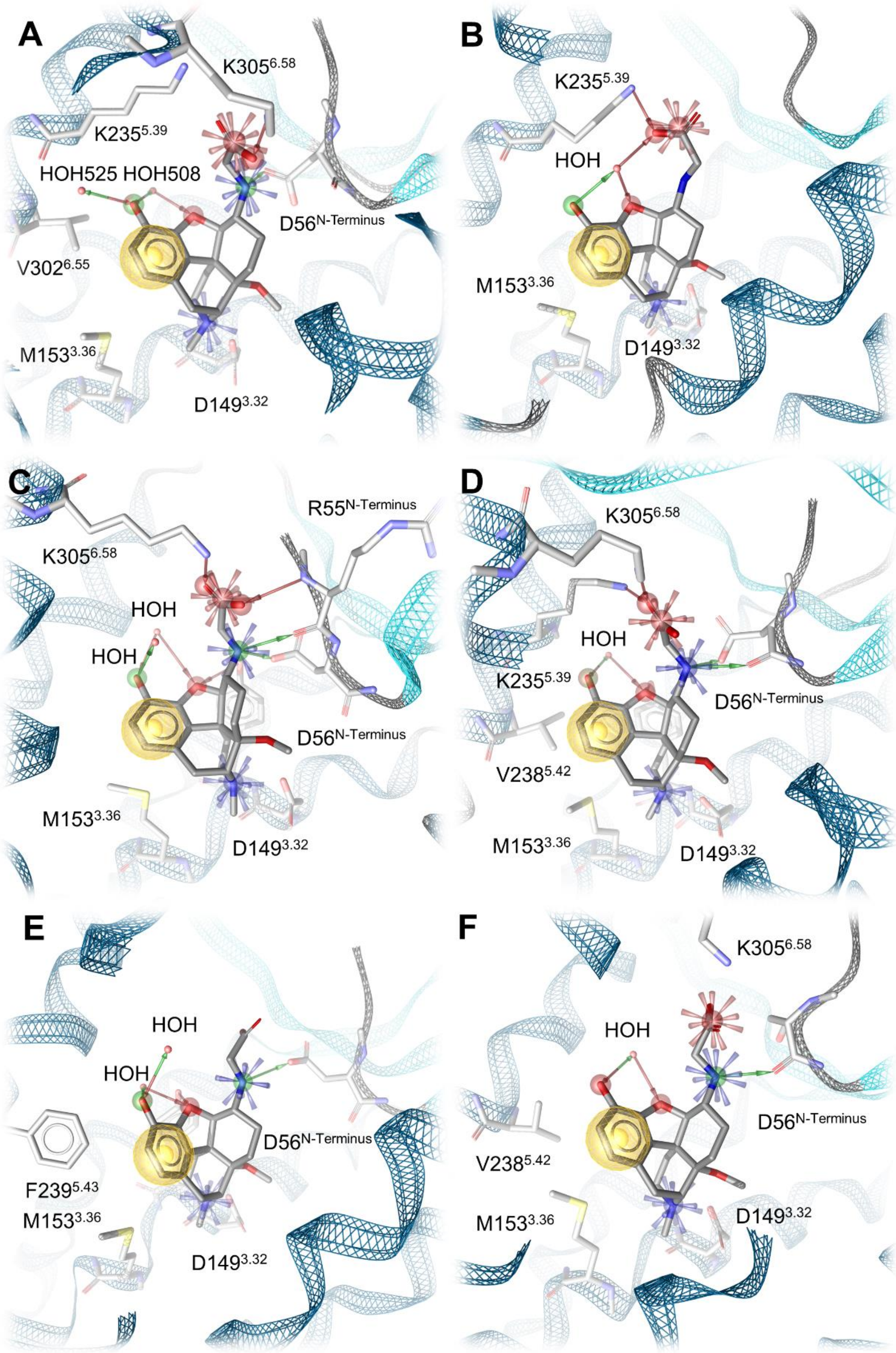

Figure S9. Comparison of the binding modes of HS-731 at the MOR derived by docking (A) and after molecular dynamics (MD) simulations (B-F). The order of the five MD simulation replicas is the following: (B) MD0, (C) MD1, (D) MD2, (E) MD3, and (F) MD4.

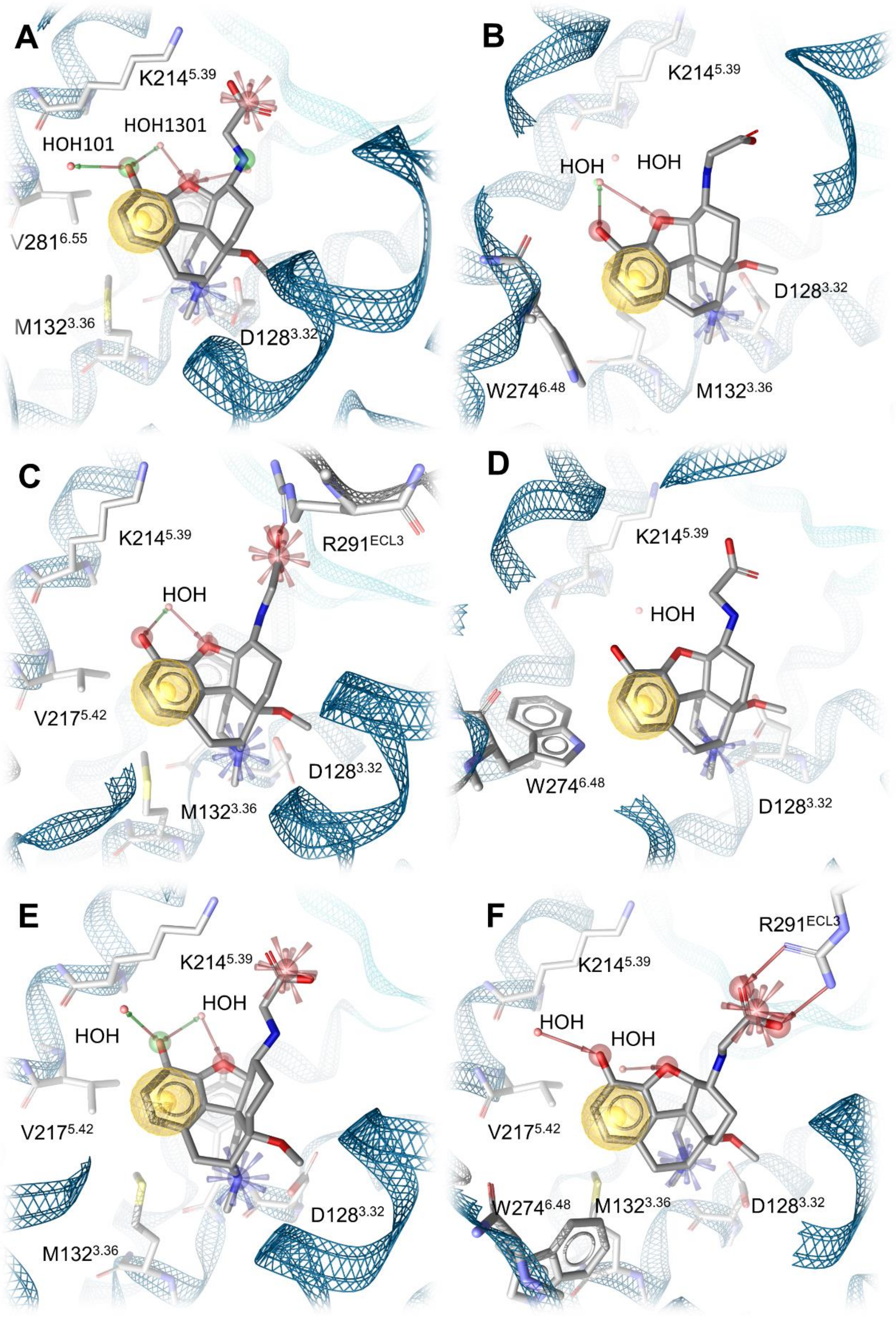

Figure S10. Comparison of the binding modes of HS-731 at the DOR derived by docking (A) and after molecular dynamics (MD) simulations (B-F). The order of the five MD simulation replicas is the following: (B) MD0, (C) MD1, (D) MD2, (E) MD3, and (F) MD4.

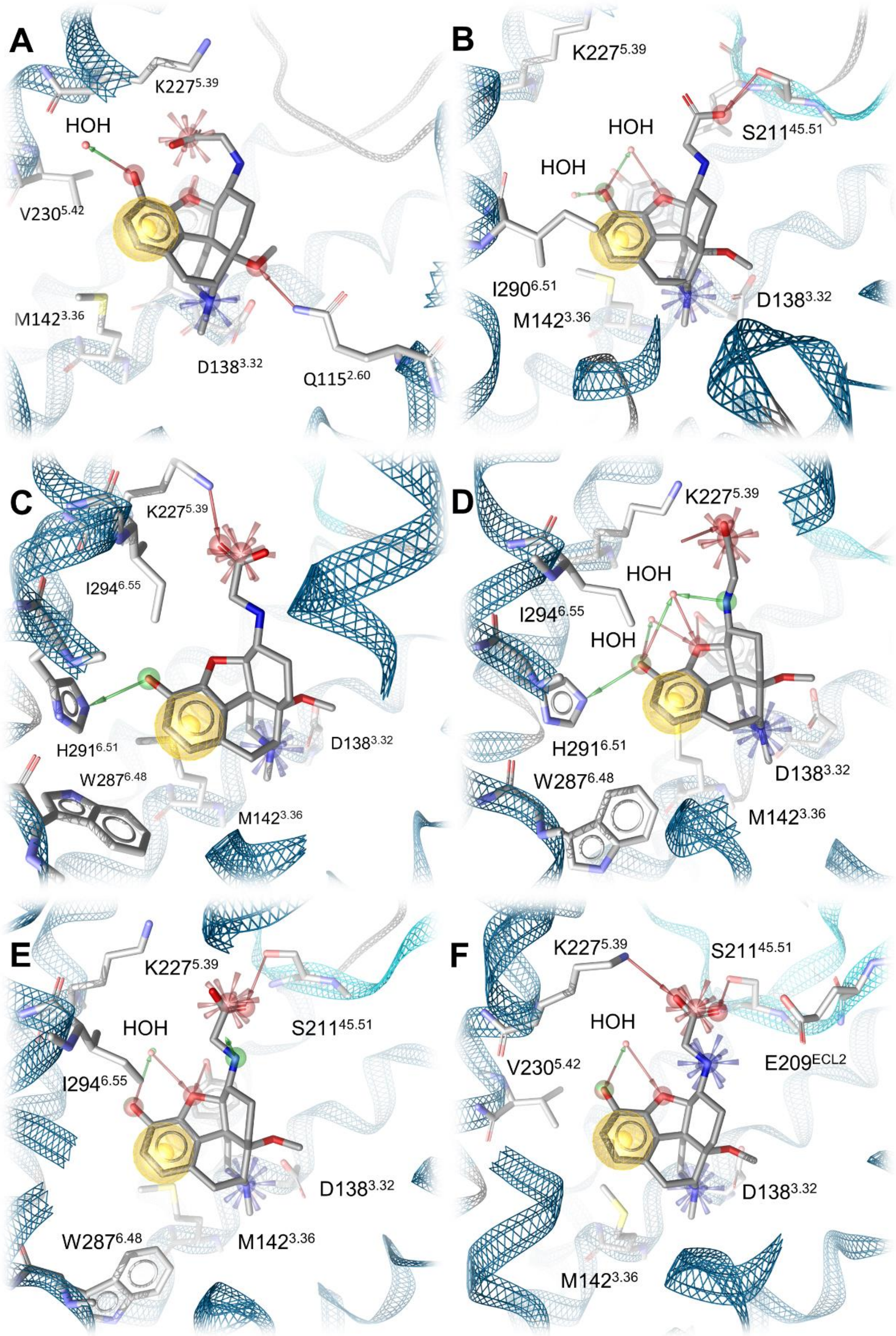

Figure S11. Comparison of the binding modes of HS-731 at the KOR derived by docking (A) and after molecular dynamics (MD) simulations (B-F). The order of the five MD simulation replicas is the following: (B) MD0, (C) MD1, (D) MD2, (E) MD3, and (F) MD4.

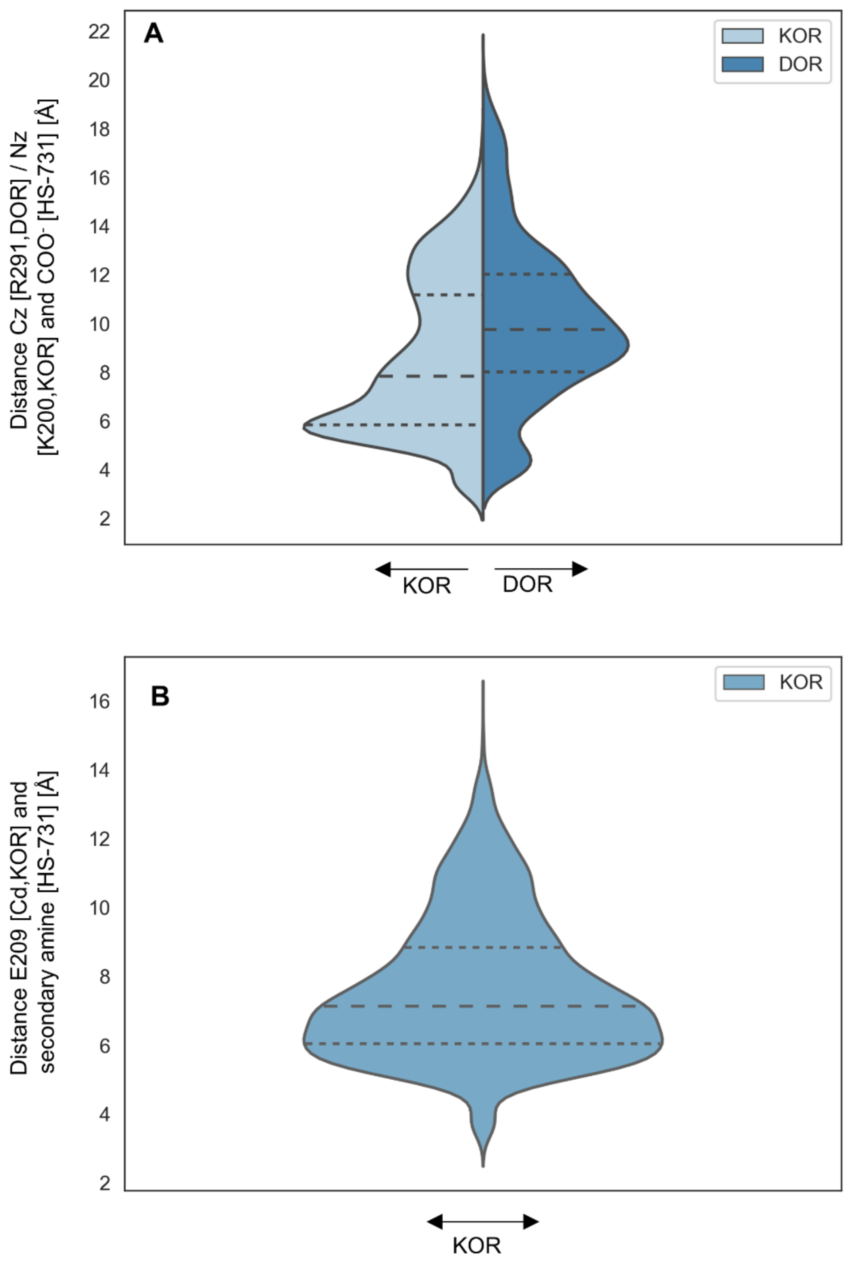

Figure S12. Ionic interaction distances. (A) Distances between $R291^{ECL3}$ (Cz, DOR) or $K200^{ECL2}$ (KOR) and the carboxylate moiety of HS-731. (B) Distances between E209ECL2 (Cd, KOR) and the secondary amine of HS-731. Dashed lines represent quantile. The width of the plot corresponds to the frequency of the measured distance.

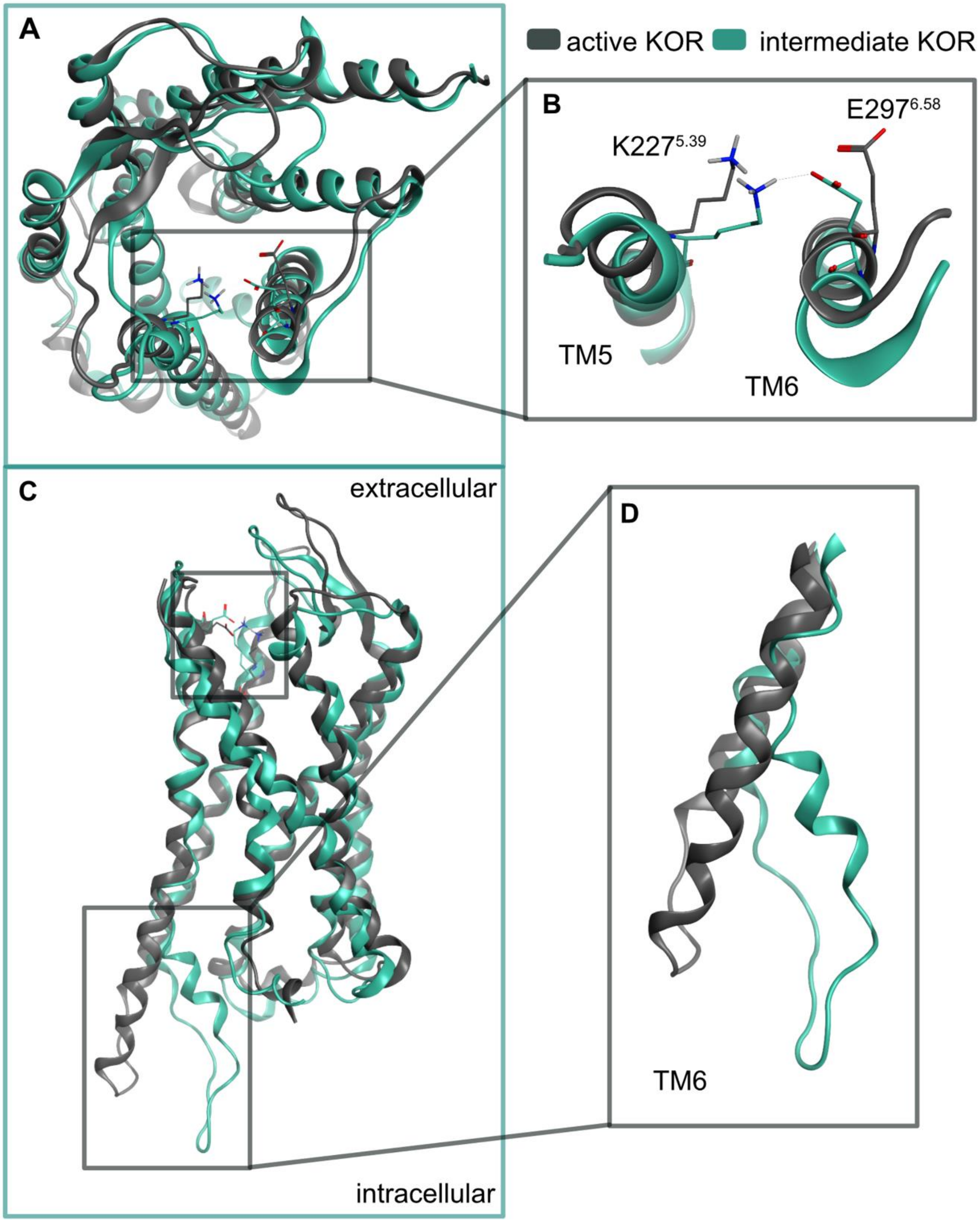

Figure S13. Comparison between the active state KOR (PDB-ID: 6B73, grey) and the intermediate state KOR (turquoise). An intermediate state KOR conformation shown is an example of an ensemble of intermediate state conformations. The intermediate state is characterized by a decreased TM6 outward movement. A) Extracellular view. B) Interaction between E297$^{6.58}$ and K227$^{5.39}$. An ionic interaction is only detectable in the intermediate state KOR but not in the active state KOR. C) Transmembrane view. Upper square depict the location of the ionic interaction also shown in B. D) The intermediate state KOR shows a pronounced inward movement of TM6 while the active state KOR exhibit a TM6 outward movement.
